# Alpha-1 antitrypsin deficiency as a common treatable mechanism in chronic respiratory disorders and for conditions different from pulmonary emphysema? A commentary on the new European Respiratory Society statement

**DOI:** 10.1186/s40248-018-0153-4

**Published:** 2018-10-08

**Authors:** Andrea Gramegna, Stefano Aliberti, Marco Confalonieri, Angelo Corsico, Luca Richeldi, Carlo Vancheri, Francesco Blasi

**Affiliations:** 1 Department of Pathophysiology and Transplantation, University of Milano; Internal Medicine Department, Respiratory Unit and Adult Cystic Fibrosis Center, Fondazione IRCCS Ca’ Granda Ospedale Maggiore Policlinico, Milano, Italy; 2 Department of Medical Sciences, Respiratory Diseases Unit, University Hospital of Cattinara, Trieste, Italy; 3 Department of Internal Medicine and therapeutics, Division of Respiratory Diseases, IRCCS Policlinico San Matteo Foundation, University of Pavia, Pavia, Italy; 4 Università Cattolica del Sacro Cuore, Fondazione Policlinico Universitario Agostino Gemelli IRCCS, Rome, Italy; 5 Regional Referral Centre for Rare Lung Diseases, Department of Clinical and Experimental Medicine, University of Catania, University–Hospital Policlinico “G. Rodolico”, Catania, Italy

**Keywords:** Alpha-1 antitrypsin deficiency, Bronchiectasis, COPD, ERS statement

## Abstract

**Background:**

The European Respiratory Society recently published an important statement reviewing available evidence on diagnosis and treatment of lung disease associated to alpha-1 antitrypsin deficiency (AATD). Several issues on this topic still remain unresolved and subject of interpretation according to different standard procedures and healthcare systems worldwide. The purpose of this commentary is to offer a critical contribution to most of these controversial issues in light of an Italian perspective for the management of this disease.

**Main body:**

The clinical spectrum of AATD lung disease might include different manifestations and the traditional paradigm of a younger emphysematous patient has been revealing insufficient. Targeting with appropriate testing only COPD patients might be considered a limited approach leading to underestimation of the real prevalence of the disease. Several reports have suggested the association between AATD and other chronic respiratory conditions, as asthma and bronchiectasis. A deeper evaluation of clinical, radiological, microbiological and functional variables is, therefore, needed in order to investigate different phenotypes in AATD patients. In addition, a new line of translational research in AATD might focus on the development of personalized therapeutic regimens taking into account the patient clinical profile and needs.

**Conclusions:**

Over the past years, AATD has been interpreted as a common mechanism of inflammatory disequilibrium and tissue damage across different conditions. Future research is gradually pointing toward this new paradigm by expanding the evidence of the role of AAT as a potent immunomodulatory and anti-inflammatory drug in conditions different from pulmonary emphysema.

## Background

Alpha-1 Antitrypsin Deficiency (AATD) is the most common hereditary disorder in adults [1]. There are several mutations of the SERPINA1 gene, encoding for AAT which is the most prevalent protease inhibitor in serum, that can cause disease. Despite its name, the major biological role of AAT is to inhibit neutrophil elastase (NE), a proteolytic enzyme targeting elastin and other basal membrane and matrix components [2]. AATD is inherited as an autosomal codominant disease and more than 100 genetic variants have been described so far and the homozigous genotype for Z allele (PI*ZZ) accounts for the vast majority of clinically recognized severe deficiency [3]. Severe AATD is a multi-organ disorder that might be associated with a large spectrum of lung disease with resulting FEV_1_ decline and impairment in gas transfer. Although the clinical course is not fully explained, patients with AATD have increased mortality, especially in the presence of smoking and severe FEV_1_ impairment [4–6]. Over the past years, a few randomized clinical trials have reported augmentation therapy with intravenous purified human AAT concentrate to improve AAT blood levels and reduce FEV_1_ or, as recently described, lung density decline in severe AATD patients [1, 7–9].

In 2017 the European Respiratory Society (ERS) published a renewed statement on diagnosis and treatment of pulmonary disease in AATD [10]. This document was developed by a task force including experts in AATD patient management, basic and clinical researchers and one methodologist. The previous 2003 ATS/ERS statement was used as a starting point [1]. The panelists addressed the most relevant issues on diagnosis, clinical management and treatment of pulmonary disease in AATD patients by the formulation of a priori questions and answered through a comprehensive search of literature. The search was limited to severe AATD cohorts (i.e. homozygous Z or null genotype and Z/null genotype). A GRADE approach to degree recommendations according to the quality of evidences was not adopted. A summary of ERS statements is reported in Table 1.

**Table 1:** Summary of European Respiratory Society statements on diagnosis, clinical management and treatment of pulmonary disease in α1-antitrypsin deficiency [10]

AATD and lung disease	• The clinical impact of AATD is highly variable. Heterogeneity in lung disease is only partly explained by exposure to known risk factors, such as cigarette smoke.
	• Lung disease in AATD generally presents at a younger age than “usual” COPD and may be misdiagnosed as asthma.
	• Although the patients’ clinical phenotype may vary they are more likely to have basal emphysema than patients with usual COPD.
	• The WHO recommends all patients with a diagnosis of COPD or adult-onset asthma should be tested for AATD.
Laboratory diagnosis and hierarchy of testing	• The quantitative determination of AAT levels in blood is a crucial first test to identify AATD. Quantitative deficiency must be supported by qualitative tests to identify the genetic mutation(s) causing AATD.
	• Protein phenotyping by isoelectric focusing identifies variants where AAT is present in the sample including the rarer variants F, I and P etc.
	• Genotyping allows a rapid and precise identification/exclusion of S and Z alleles and other variants, where specific primers are available.
	• Gene sequencing remains necessary for those cases where a null variant or a deficient variant other than Z or S is suspected.
	• Testing of relatives of identified patients should be considered after appropriate counselling.
	• Genetic testing should be carried out only after informed consent is given and in accordance with the
	relevant guidelines and legislation.
Lung disease progression in AATD	• Annual measurement of lung function including post-bronchodilator FEV1 and gas transfer provides information about disease progression.
	• Lung densitometry, as performed in observational cohort studies and randomised clinical trials is the most sensitive measure of emphysema progression.
	• The correlation between change in lung density and any short-term change in measures of pulmonary function is weak. However, in the longer term, CT lung density decline correlates with reduction in FEV1 and health status.
	• The role of CT in the follow-up of patients in routine clinical practice requires further validation.
The risk of lung disease in heterozygotes	• Never-smoking PiMZ subjects do not have an increased risk for COPD.
	• Smoking PiMZ and PiSZ subjects have an increased risk of COPD compared to smoking PiMM subjects.
	• The role of other heterozygotes remains unknown due to their rarity and potential ascertainment bias from measuring AAT in unusual cases of lung or liver disease.
Role and benefits of screening	• Most screening studies have been biased as they did not involve random population samples.
	• Population-based screening studies provide less biased prevalence estimates of specific AATD protein and clinical phenotypes as well as valuable insights into the natural history of AATD.
	• Neonatal screening has been shown to be effective in reducing the smoking rates for 18–20-year-olds compared to age-matched individuals.
	• Screening may have negative psychological effects on parents and on mother–child bonding. However, these negative effects can be addressed by comprehensive genetic counselling and care provision at centres of excellence for AATD.
Augmentation therapy for AATD	• Several randomised clinical trials in severe AATD have shown intravenous augmentation therapy to reduce the progression of emphysema as assessed by CT densitometry.
	• There is no evidence to support efficacy of AAT augmentation therapy in PiSZ, PiMZ or current smokers of any protein phenotype.
	• Clinical trials have used fixed doses of AAT determined by body weight. Whether individualising dosage based on trough levels for each patient has any benefit requires confirmation.
Lung volume reduction surgery in AATD	• Surgical volume reduction and EBV placement may be considered in selected patients with AATD, but further studies are needed to confirm the role of such therapies.
	• The optimal results of these techniques are obtained when a careful appraisal of risks and benefits are performed by a multidisciplinary team experienced in LVR and AATD.
Lung transplantation for emphysema associated with AATD	• The survival benefit of lung transplant in AATD patients is not clear.
	• In general, patients with AATD have improved quality of life following lung transplantation.
	• Referral timing, rate of decline in lung function, health status and social support differ from patient to patient, and will have an influence on the evaluation for transplant.
	• The role of post-transplant augmentation therapy in particular needs to be explored.
New lines of research in AATD	• According to the European Council, management of patients with AATD should be supervised by reference centres of excellence at a national or regional level.
	• The systematic collection of data concerning clinical characteristics and natural history of patients with AATD in national and international registries will enhance knowledge about the evolution of this disease and its optimal management.
	• For many AATD individuals a respiratory service is the first point of diagnosis. The operational pathway includes varying assessments and follow-up depending on personalising the patients’ risk and defining the respiratory phenotype. Links to multidisciplinary teams will ensure the best quality of care.

The purpose of this commentary is to offer a contribution to most controversial issues regarding pulmonary disease associated with AATD. In presenting these arguments, three main topics will be addressed, including 1) the limitation of the current definition of AATD lung disease; 2) the need of broader testing and improvement of AATD awareness among physicians taking care of chronic respiratory diseases; 3) new paradigm and perspectives in AATD future research.

## Heterogeneity of AATD associated lung disease

In the attempt to give a definition of associated lung disease, the task force attributed a wide degree of heterogeneity to pulmonary involvement in AATD. However, in their considerations authors relied on the classical phenotype of a patient with onset of COPD at a younger age and more likely to have basal panlobular emphysema than usual COPD patients [10]. This is, in our view, a crucial point.

AATD pulmonary involvement might include emphysema with severe functional and radiological impairment as well as different pulmonary pictures or, even, normal chest CT scan [11]. In a series of PI*ZZ patients, Gishen analyzed 165 chest radiographs and reported emphysema pattern only in 20% of patients, while 15% appeared completely normal [12]. DeMeo and colleagues demonstrated in a population of 378 patients with severe AATD (PI*ZZ) that variability in FEV_1_ was huge and did not relate to smoking status or younger age. Thus, some smokers had preserved lung function, while some non-smokers had lower FEV_1_ [13]. These data suggest that neither FEV_1_ nor chest radiology are sufficient to rise clinical suspicion of AATD in daily practice. As a consequence, most of severe AATD patients might not be still identified and case finding only among COPD patients is not sufficient. In addition, the unusual association between most common deficitary alleles in AATD and idiopathic pulmonary fibrosis (IPF), or combined pulmonary fibrosis and emphysema (CPFE) have also been described [14, 15].

**Fig. 1:**
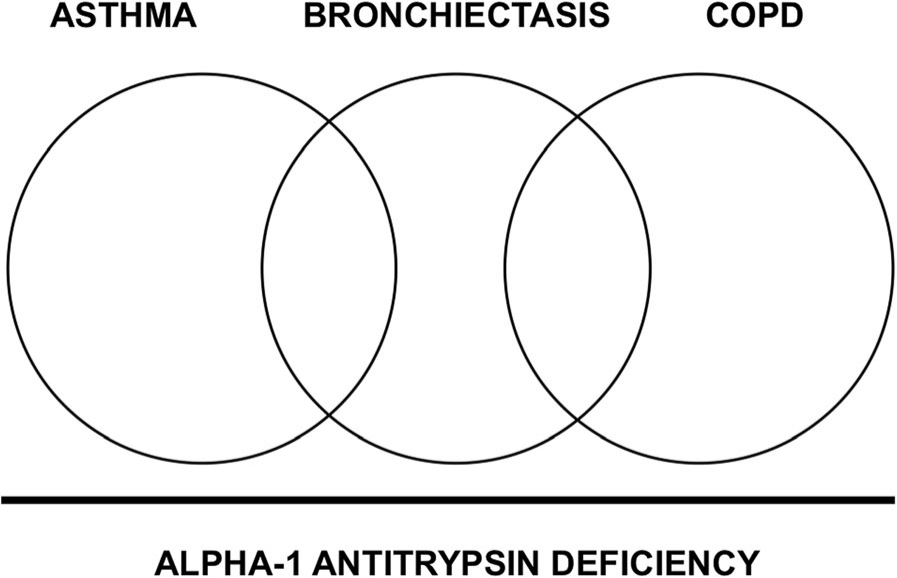
AATD might be interpreted as a common mechanism with different clinical manifestations and frequent overlap among chronic respiratory disorders

Targeting with appropriate testing only those patients with classic AATD clinical presentation might be considered a limited approach leading to underestimation of the real prevalence of the disease. In view of the extreme variety of this condition, the paradigm of pure emphysematous patients has gradually changed by the understanding that the clinical spectrum of AATD lung disease might include different phenotypes (Fig. 1).

### AATD and asthma

A review explored the association and overlap between AATD and asthma [16]. Subsequent to the first descriptions of AATD patients, Makino and Black reported case series of emphysematous patients with clinical feature of airway reactivity and wheezing [17, 18]. In the next years, several reports ranged the prevalence of asthma in AATD population from 4 to 34% [19–22]. In the National Heart, Lung and Blood Institute’s Registry of AAT Deficient Individuals (NHLBI), a network of 37 centers across USA and Canada for a total of 1219 subjects, 55% of patients reported a significant response to bronchodilation, while concomitant asthma and respiratory allergy were reported in 31% and 23% of cases, respectively [23, 24]. On this basis, in 1997 the WHO recommended that all patients with COPD and adult-onset asthma should be screened for AATD [25]. A recent study conducted through the Alpha-1 Foundation Research Registry, involving 500 patients with severe deficiency, confirmed that clinical manifestations of uncontrolled asthma or asthma with fixed obstruction are frequent in AATD; however, among the 34% participants undergoing allergological evaluation, only 5% were diagnosed with AATD [26].

Although current literature is controversial about the prevalence of AAT deficiency alleles in asthmatic population, AATD was reported in 2.4% of patients with poorly controlled asthma with 10.5% being a carrier of a deficiency mutation [27, 28]. This contributed to the interpretation that AATD itself might predispose to airway hyper-reactivity and participate to asthma pathophysiology. While the underlying biological mechanism is not understood, data from basic research have in fact suggested that AAT has also immunomodulatory functions and might affect eosinophilic cells [29].

Finally, AATD patients with concomitant asthma have worse prognosis. Bronchodilator response has been associated with greater FEV_1_ decline and poorer clinical outcomes [13, 24, 30]. From the clinician’s point of view, improving diagnosis in this population is fundamental to optimize clinical management and further clinical trials are needed to investigate patients’ response to available treatments.

### AATD and bronchiectasis

Several reports have suggested the association between AATD and bronchiectasis [21, 31, 32]. In order to address the prevalence and clinical impact of bronchiectasis in this population, Parr and Stockley retrospectively studied 74 subjects with severe AATD who had received a HRCT scan in the time period 1995–2002. They reported an unexpectedly high prevalence of bronchiectatic changes in almost all patients (70/74 subjects, 94.6%) without regard to clinical manifestations. If criteria for clinically significant bronchiectasis were applied, i.e. chronic cough with sputum production in association with bronchial dilation in at least four bronchial segments, bronchiectasis occurred in 27% of study population [33].

The concept of association between bronchiectasis and AATD is also supported by data coming from a large targeted screening program for AATD in Germany. Greulich and colleagues analyzed 18,638 testing kits and identified 1835 patients with severe AATD (9.82% of the tested population). Along with emphysema and COPD, bronchiectasis demonstrated to be a strong predictor for PI*ZZ genotype suggesting the implementation of screening in this emerging population [34].

From a different perspective, among physicians taking care of bronchiectasis AATD has been recognized as a cause of bronchiectasis and a causal link has been hypothesized. The common origin of airway disease in AATD might be explained by the disequilibrium between neutrophilic inflammation and protease inhibitors, thus resulting in bronchial connective tissue damage [35]. In addition, AAT has been demonstrated to act as an anti-inflammatory mediator by the inhibition of TNF-α and IL-1β levels in mononuclear cell cultures [36]. However, whether bronchiectasis comes from a primary mechanism of the disease or is the result of recurrent respiratory infection is still matter of debate.

In 2015 Lonni and co-authors conducted an extensive analysis of seven cohorts of adult outpatients with bronchiectasis prospectively enrolled between 2009 and 2013 in different countries across Europe [37]. All patients underwent the same etiological workup as suggested by BTS guidelines, including quantitative analysis of AAT in plasma in those subjects with radiological emphysematous changes. AATD was identified as a cause of bronchiectasis in a total of 8 (0.6%) of population. This result is consistent with those reported by a comprehensive review performed to identify papers reporting the underlying etiologies of published cohorts; AATD was identified as a cause of bronchiectasis in 36 (0.4%) out of 8608 patients [38]. The vast majority of those patients came from Europe (*n* = 23) and Northern America (*n* = 12), suggesting that only a portion of studies performed in developed countries had provided some sort of testing on AATD.

Over the past decade, different national and international registries have been set up to evaluate the real-life characteristics of patients with different respiratory diseases, including bronchiectasis, severe asthma and NTM [39] https://www.registroirene.it/it/user [40]. Looking into the Italian situation, the first data from the Italian Registry on Adult Bronchiectasis (IRIDE) have been presented at the 2nd World Bronchiectasis Conference http://www.world-bronchiectasis-conference.org/wp-content/uploads/2017/07/AbstractBook_WEB.pdf. Among 522 adults with bronchiectasis (to May 2017) the diagnosis of AATD was less than 1% of population.

Although this amount of data documented the role of AATD as an underlying etiology of bronchiectasis, it is possible to presume that a majority of patients never received AAT testing. In 2014 the Italian Respiratory Society (IRS/SIP) conducted a national audit on adult patients with bronchiectasis attending secondary care clinics in Italy [41]. The audit analysed adherence among physicians to quality standards as suggested by the British Thoracic Society. To date, this represents the only testing of quality standards for bronchiectasis in Italy. In regard to etiological screening of bronchiectasis, only 8.2% of patients were tested for AATD. As a result, it is likely to hypothesize that estimation of AATD prevalence in bronchiectasis population is poor.

Notably, both in the European analysis as well as in the Italian report COPD was among the three most prevalent cause of bronchiectasis with 15% and 7.8% of population, respectively [37] http://www.world-bronchiectasis-conference.org/wp-content/uploads/2017/07/AbstractBook_WEB.pdf. The association between COPD and bronchiectasis might represent a distinct phenotype in patients characterized by chronic respiratory infections, more severe airway inflammatory pattern and frequent pulmonary exacerbations [42]. However, the question if COPD and bronchiectasis co-exist as two indipendent diseases or one is the cause of the other still remains unanswered.

### AATD and NTM

Nontuberculous mycobacteria (NTM) are a large number of pathogenic and non-pathogenic mycobacterial species different than *m. tubercolosis* complex. Recent reports demonstrated an increasing role of NTM in patients with underlying respiratory disorders, as bronchiectasis and COPD [43–45]. However, susceptibility to the development of NTM pulmonary disease is different among patients and AATD has been postulated as a potential predisposing factor. In a cohort of 100 patients with NTM pulmonary disease, AAT genetic variants were reported in 27% of cases, which is 1.6 times than estimated prevalence in general population. Most of those patients showed Pi*MS and Pi*MZ heterozigosity and no sign of pulmonary emphysema. In addition, AAT has also been demonstrated to impair *M. abscessus* phagocytosis by human macrophages, thus forbidding NTM the ideal intracellular milieu [46]. Thus, in consideration of its pleiotrophic anti-inflammatory and immunomodulatory effects, AATD might increase patient vulnerability to NTM both directly and through bronchiectasis development.

## Clinical implications: The role and benefits of screening

Since a majority of AATD patients is still unidentified and delays in making the correct diagnosis are common, structured programs of AAT testing have been discussed. ERS statement discussed the role and benefits of screening for AATD and summarized current evidence (Table 1). Most of population screening of adults in the past has been limited to specific groups, as blood donors, with resulting esteem of prevalence biased by selection [10]. A more accurate evaluation came from programs including random population samples [41, 42]. A large population-based screening was performed in Ireland on 3000 subjects and identified 42 ZZ, 44 SZ and 430 MZ individuals, a prevalence estimation greater than previously thought [47]. Another experience came from Poland where 859 adult subjects (mean age 49.5 years, range 20–90) were screened with resulting frequencies of PI*S and PI*Z equal to 17.5 per 1000 and 10.5 per 1000, respectively. Therefore, the estimated prevalence of AATD was high enough to consider implementation of a large-scale screening program [48]. However, the ERS statement reported that no RCT investigated efficacy and effectiveness of AATD screening programs and evidence for this approach is currently poor [10].

This bundle of evidence has been evaluated differently across guidelines regarding chronic respiratory condition that might be related to AATD. Although AATD is strongly associated to the development of COPD, The Global Initiative for Chronic Obstructive Lung Disease (GOLD) document edited in 2018 mentioned AATD in only two occasions. First, as a known genetic risk factor for COPD and an example of gene-environment interaction; second, as a step in COPD assessment that WHO guidelines recommended to screen once in all patients with a diagnosis of COPD, especially in areas with high AATD prevalence [49]. On the contrary, Global Initiative for Asthma Management and Prevention (GINA) document has only one mention of AATD as a possible differential diagnosis in patient with suspected asthma in presence of family history of emphysema and aged between 12 and 39 years. Notably, no indication for AATD testing in asthmatic patients can be found in this document [50].

In regard to bronchiectasis, the scenario is more complex since international guidelines did not exist until recently. A few national guidelines did not recommend routine AATD testing unless the radiological investigations suggest basal emphysema [51, 52]. Otherwise, guidelines from Pulmonology Portuguese Society included AAT quantitative test in the minimum bundle of etiological screening for all patients with bronchiectasis [53]. In 2017 the ERS published the first international standards of care for bronchiectasis in adult patients [54]. This document suggests providing AATD testing only in the presence of basal emphysema or early onset airway obstruction. A comprehensive summary of recommendations for AATD testing in different documents is provided in Table 2.

**Table 2:** A comprehensive summary of indications for AATD testing across documents for the clinical management of chronic respiratory disease

Document	Disease	Indications for AATD testing
Global strategy for prevention, diagnosis and management of COPD, 2018 [49]	COPD	All patients with a diagnosis of COPD, especially in areas with high AATD prevalence
Global strategy for asthma management and prevention, 2018 [50]	Asthma	No indication for AATD testing
European Respiratory Society, 2017 [54]	Bronchiectasis	Patients with basal emphysema or early onset airflow obstruction
Pulmonology Portuguese Society Bronchiectasis Study Group, 2018 [53]	Bronchiectasis	All patients with a diagnosis of BE
Thoracic Society of Australia and New Zealand, 2015 [62]	Bronchiectasis	No indication for AATD testing
British Thoracic Society, 2010 [51]	Bronchiectasis	Patients with basal emphysema.
Normativa SEPAR, 2008 [52]	Bronchiectasis	Patients with emphysema and /or liver disease

This lack of common ground in testing AAT across respiratory diseases needs further considerations. Identifying AATD is fundamental in view of clinical, social and psychological consequences in target population. First, AATD is a systemic disease with extra-respiratory clinical manifestations that might benefit from a specific management with positive impact on clinical outcomes and patients’ quality of life. AATD patients should have access to reference centers, that are able to provide the best standard of care and contribute to clinical data networking. Furthermore, patients affected by a rare disease might benefit from free care or proper health insurance issues across different jurisdictions. From a psychological perspective, AATD is both a genetic disease with increased risk of affected children and a chronic condition with possible impact on family planning and patients’ life priorities. In this respect, AATD patients should be provided with proper genetic counseling and support.

### Different perspectives of AATD definition and indications for augmentation therapy

The definition of AATD might rise some controversial among physicians in respiratory care. Three different scenarios are possible: 1) deficiency as the condition of lower-than-normal AAT levels in blood; 2) deficiency as the result of mutations affecting SERPINA gene; 3) deficiency as the indication for augmentation therapy for AATD.

*AAT deficiency as the condition of lower AAT levels in blood.* This definition is focused on the quantitative determination of blood levels and represent the widest interpretation of deficient status. Currently nephelometry and immunoturbidimetric assay are the most widely employed techniques; in this regard, ERS statement reported a comprehensive list of laboratory methodologies used in central laboratories across Europe [10].

### AAT deficiency as the result of a genetic disease

As suggested by ERS statement, abnormal quantitative measurements of AAT levels in blood must be supported by a qualitative test detecting SERPINA gene mutations on both copies of the gene [7]. The normal allele, designated as M, is present in 85–90% of population, while the prevalent deficient alleles are designated as S and Z [1]. As a consequence, the most common genotypes result from the combination of these allelic forms: the homozygous genotype Pi*MM (wild-type), the heterozygous Pi*MZ and Pi*MS and the semi-deficient or deficient genotypes Pi*SZ, Pi*SS and Pi*ZZ. Besides deficient alleles S and Z, about 50 other uncommon mutations have been described, including (null) allele and other rare variants [55].

### AAT deficiency as the indication for augmentation therapy

Intravenous infusion of alpha1-proteinase inhibitor, prepared from pooled human plasma of normal donors, has the biochemical goal to raise and maintain serum AAT levels above the protective threshold and clinical goals to slow the progression of emphysema, reduce number of exacerbations and enhance the duration and quality of life. [56, 57].

Up to now augmentation therapy has been approved only for commercial use in adults with severe AAT deficiency-related pulmonary emphysema and a number of AAT preparations are currently available in various markets [56].

Evidence that augmentation therapy brings benefits (eg, slowed rate of FEV1, decline and decreased mortality) is stronger for individuals with moderate obstructive pulmonary impairment (eg, FEV1 35–60% predicted) than for those with severe airflow obstruction [57].

## Implications for future research

In consideration of the bundle of evidence showing a causative role of AATD in bronchiectasis, a deeper evaluation of clinical, radiological, microbiological and functional variables should be applied in order to investigate different phenotypes in AATD patients. The identification of a distinct bronchiectasis phenotype might be useful for a better understanding of AATD pathophysiology, as well as to improve personalized approach towards sub-groups of patients that might benefit from a focused treatment. A new generation of RCTs is, therefore, needed to test efficacy of intravenous AAT augmentation therapy in patients with combined bronchiectasis and severe AATD. At the same time, new end-points for monitoring progression of lung disease and response to therapy in this population ask to be explored.

**Fig. 2:**
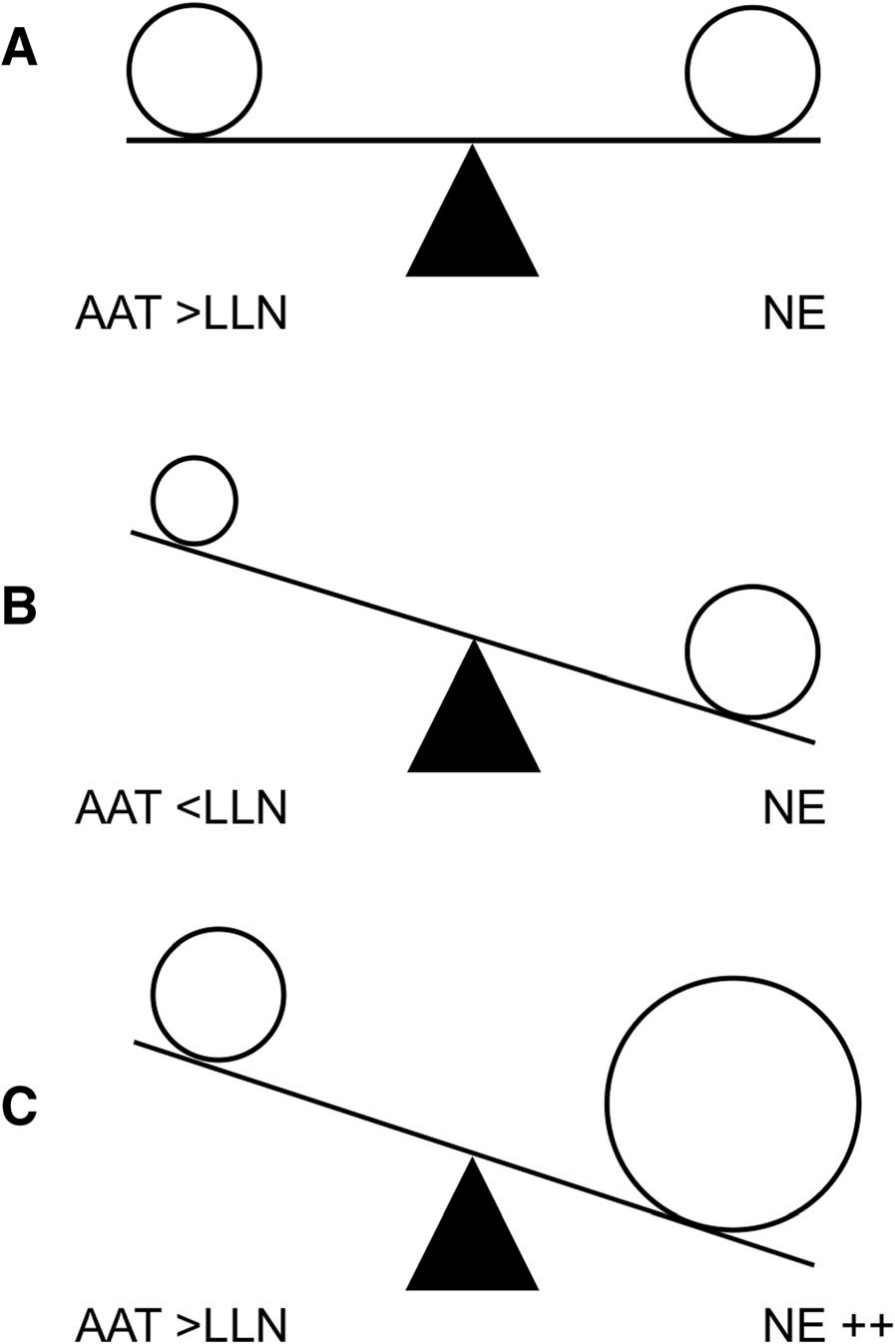
The protease/anti-protease balance in healthy subjects (**a**), AATD patients (**b**) and bronchiectasis patients (**c**). Definitions. AAT = alpha-1 antitrypsin deficiency; NE = neutrophil elastase; LLN = lower limit of normality

In addition, decisions for starting the chronic augmentation therapy need to be made at this point on a risk/benefit basis, as the lung destruction in emphysema is irreversible and a new line of translational research in AATD might focus on the development of personalized therapeutic regimens taking into account the patient clinical profile and needs [58]. In this light, investigating a new role for augmentation therapy in patients with heterozygosity (PI*MZ, PI*MS) is urgently needed, especially in the presence of other chronic respiratory conditions as asthma or bronchiectasis. The rationale is that the anti-protease activity provided by AAT, although present in normal amounts, is finally compromised in patients with chronic inflammatory disease, such as bronchiectasis, due to the overwhelming NE inflammatory burden [2]. The condition would result in functional deficiency of AAT (Fig. 2). This supports the idea of an individualized approach to augmentation therapy based, not only on the genotype of patient, but also on a careful evaluation of expected benefits, risks and sanitary costs.

The use of AAT treatment in patients other-than-PI*ZZ dates back decades and has been already explored in cystic fibrosis (CF), a genetic systemic disease characterized by massive neutrophilic inflammation on the lung epithelial surface. In 2007 Griese and colleagues performed a prospective uncontrolled study of 4-week inhaled AAT in a cohort of 52 patients with CF. The primary outcome of this study was a reduction in NE activity, along with change in neutrophils and pro-inflammatory cytokines on sputum. Although results on pulmonary function were controversial, the authors found a significant decrease in all inflammatory markers measured during treatment [59]. Similarly, two other studies showed reduction in elastase activity in CF subjects following inhaled treatment of AAT [60, 61]. However, a correlation with clinical outcomes would require long-term multicentre studies.

## Conclusions

During the last years AATD has been interpreted as a common mechanism of inflammatory disequilibrium and tissue damage among both chronic respiratory diseases and extra-respiratory conditions. The final clinical picture would, therefore, result of a complex and mostly unknown interaction between genetic pattern and environmental factors. Future research is gradually pointing toward this new paradigm by expanding the evidence of the role of AAT augmentation treatment as a potent immunomodulatory and anti-inflammatory drug for conditions different from pulmonary emphysema (Table 3).

**Table 3:** A list of last decade clinical trials testing AAT safety and efficacy in conditions different from severe deficiency AAT according to clinicaltrials.gov, last access 15 july 2018

Study Title	Condition	Phase	Start Date	Status
A proof-of-concept pilot trial of AAT for pre-emption of steroid refractory acute GVDH	GVDH	2	2018	Recruiting
Multicenter trial of the effect of AAT on Islet Transplant engraftment and durability after Renal Transplant	Diabetes Mellitus, Type 1	2	2017	Recruiting
Anti-inflammatory therapy to improve outcomes in patients with Chronic Pancreatitis undergoing Total Pancreatectomy Islet Autotransplantation	Chronic Pancreatitis; Diabetes Mellitus, Type 1	4	2016	Recruiting
AAT enhances islet autograft survival	Chronic Pancreatitis	1–2	2016	Recruiting
A phase 2/3 clinical study to evaluate the safety and efficacy of AAT as an add-on biopharmacotherapy to conventional steroid treatment in subjects with acute GVDH with lower gastrointestinal involvement	GVDH	2–3	2016	Terminated
A proof-of-concept study evaluating the safety and efficacy of human AAT treatment in first Lung Transplantation	Transplantation, Lung Rejection	2	2015	Active, not recruiting
Improving single donor success rate in clinical islet using AAT	Diabetes Mellitus, Type 1	1–2	2015	Active, not recruiting
A multicenter, randomized, placebo-controlled study to evaluate the safety and efficacy of a human plasma-derived AAT in subjects with new-onset Type 1 Diabetes Mellitus	Diabetes Mellitus, Type 1	2	2014	Terminated
Single dose administration of AAT for the amelioration of organ injury and post-operative bleeding in patients undergoing cardiac surgery with cariopulmonary bypass	Post Cardiac Surgery Systemic Inflammatory Response	1–2	2014	Unknown
Pilot study of AAT: a novel treatment to mitigate Neuromyelitis Optica attacks	Neuromyelitis Optica	1	2014	Unknown
Long-term treatment safety, tolerability and efficacy of AAT in Type 1 Diabetes	Diabetes Mellitus, Type 1	2	2013	Completed
AAT to quench the acute inflammatory response in ST-segment elevation Acute Myocardial Infarction	Acute Myocardial Infarction	1–2	2013	Completed
Phase 2 study to evaluate the efficacy and safety of human AAT in the treatment of new onset Type 1 Diabetes	Diabetes Mellitus, Type 1	2	2013	Completed
Effects of Prolastin aerosol therapy on bacterial density in the airways of patients with CF	Cystic Fibrosis	2	2013	Terminated
AAT in treating patients with acute GVDH	GVDH	1–2	2012	Completed
Safety and tolerability of inhaled AAT once-a-day in patients with Cystic Fibrosis	Cystic Fibrosis	2	2012	Completed
Safety and efficacy of AAT in HIV Disease	HIV Disease	2–3	2012	Terminated
A pilot study of AAT in steroid refractory acute GVDH	GVDH	2	2012	Active, not recruiting
The effects of AAT on the progression of Type-1 Diabetes in subjects with detectable C-peptide	Diabetes Mellitus, Type 1	1	2011	Completed
Proof-of-concept study of the safety, tolerability and efficacy of intravenous AAT in Type 1 Diabetes	Diabetes Mellitus, Type 1	1–2	2011	Completed
Safety and efficacy of inhaled AAT in preventing Bronchiolitis Obliterable Syndrome in Lung Transplant recipients	Transplantation, Lung Rejection	2	2011	Unknown
Safety and tolerability study of AAT inhalation solution in patients with Cystic Fibrosis	Cystic Fibrosis	1	2011	Completed
Effects of intravenous AAT on preserving beta-cell function in new-onset Type 1 Diabetes Mellitus	Diabetes Mellitus, Type 1	2	2010	Withdrawn

*AAT* alpha-1 antitrypsin, *CF* cystic fibrosis *GVDH* graft-versus-host disease, *HIV* human immunodeficiency virus

## Abbreviations

AATD: alpha-1 antitrypsin deficiency
COPD: Chronic Obstructive Pulmonary Disease
CPFE: combined pulmonary fibrosis and emphysema
IRIDE: Italian Registry on Adult Bronchiectasis
NE: neutrophil elastase
NTM: Nontuberculous mycobacteria
